# The clinical characteristics and short-term prognosis in elderly patients with Guillain–Barré syndrome

**DOI:** 10.1097/MD.0000000000005848

**Published:** 2017-01-10

**Authors:** Bing Zhang, Xiujuan Wu, Donghui Shen, Ting Li, Chunrong Li, Mei Mao, Hong-Liang Zhang, Kangding Liu

**Affiliations:** Neuroscience Center, Department of Neurology, the First Hospital of Jilin University, Jilin University, Changchun, China.

**Keywords:** clinical features, elderly, Guillain–Barré syndrome, short-term prognosis

## Abstract

To investigate the clinical characteristics and short-term prognosis of elderly patients with Guillain–Barré syndrome (GBS).

We retrospectively analyzed the clinical data of adult GBS. According to the age, the enrolled subjects were divided into 2 groups, that is, patients ≥60 years (elderly group) and those aged 18 to 59 years (nonelderly group). The clinical characteristics and short-term prognosis of the patients in the 2 groups were compared.

In total, 535 patients were enrolled. There were 67 patients fell into the elderly group with a mean age of 69 years old; while 468 patients fell into the nonelderly group with a mean age of 39 years old. We found that the elderly patients had significantly lower incidence of antecedent infections (49.3% vs 66.2%, *P* < 0.01). The time from onset to admission (5 vs 4 days, *P* < 0.05) and time from onset to nadir (7 vs 6 days, *P* < 0.05) were significantly longer in the elderly patients. It was noteworthy that more elderly patients were found with lymphocytopenia (55.4% vs 37.3%, *P* < 0.01), hyponatremia (25.0% vs 10.2%, *P* < 0.01), hypoalbuminemia (9.0% vs 2.6%, *P* < 0.05), and hyperglycemia (34.3% vs 15.2%, *P* < 0.01). Importantly, the elderly patients had longer duration of hospitalization (17 vs 14 days, *P* < 0.05), higher incidence of pneumonia (29.9% vs 18.8%, *P* < 0.05), and poorer short-term prognosis (58.2% vs 42.7%, *P* < 0.05). In patients with severe GBS, no significant differences were observed in disease severity, treatment modality, incidence of pneumonia, and duration of hospitalization between the 2 groups. However, more patients in the elderly group showed poor short-term prognosis (84.1% vs 63.8%, *P* < 0.01). Further, old age (≥60 years) (OR = 2.906, 95% CI: 1.174–7.194, *P* < 0.05) and lower Medical Research Council (MRC) score at nadir (OR = 0.948, 95% CI: 0.927–0.969, *P* < 0.01) were risk factors for poor short-term prognosis in severe GBS patients.

The clinical characteristics and short-term prognosis of elderly patients with GBS are distinct from nonelderly adults. Old age (≥60 years) and lower nadir MRC score serve as predictor for poor short-term prognosis in severe GBS patients.

## 1 Introduction

Guillain–Barré syndrome (GBS) is an immune-mediated disease of the peripheral nervous system. About two-thirds of GBS are triggered by antecedent infectious agents, leading to the concept that GBS is a postinfectious immune-mediated disorder; however, accumulating GBS following noninfectious factors has also been reported in recent years.^[1]^ Additionally, it was reported that transient immunosuppression may be an important link in the pathogenesis of GBS.^[2]^ GBS could occur at any age.^[3]^ The epidemiological data from western countries found that the incidence of GBS increased with age;^[4]^ however, the incidence of GBS based on the epidemiological data in the elderly patients in China showed significant differences, which found that the incidence of GBS among elderly patients was remarkably lower in Harbin while the GBS incidence increased with age in Jiangsu province.^[5,6]^ In addition, studies have shown that elderly patients with GBS have more severe disease, less involvement of cranial nerve, more axonal damage, and slower recovery; however, the results of different studies show variation.^[7–9]^ Also, some studies have demonstrated that old age was an important factor in predicting poor prognosis of GBS besides the severity of the disease.^[10–12]^ The aim of this study was to explore the clinical features and short-term prognosis of elderly patients with GBS through a retrospective study.

## 2 Subjects and methods

### 2.1 Subjects

This retrospective study was approved by the ethics committee of The First Hospital of Jilin University, Changchun, China. Subjects were selected from patients who met the diagnostic criteria of GBS^[13]^ and received sequential treatment during hospitalization in the Department of Neurology of the First Hospital of Jilin University, during January 2003 to December 2014. Subjects were excluded from the study if they: were aged < 18 years; refused the treatment or diagnosed with chronic inflammatory demyelinating polyradiculoneuropathy or Miller Fisher syndrome. The subjects were categorized into 2 groups based on their age: elderly group (≥60 years) and nonelderly group (<60 years). Clinical data from all subjects were analyzed retrospectively including age, gender, season of disease occurrence, antecedent infections (mainly include upper respiratory tract infection, diarrhea, and fever of unknown origin), initial symptoms, time from onset to admission/nadir, tendon reflex, sensory disturbances, cranial nerve damage, Medical Research Council (MRC) score, and Hughes Functional Grading Scale (HFGS) score at nadir, whether requiring mechanical ventilation, complications, treatment modality, duration of hospitalization, MRC and HFGS score at discharge, laboratory test results, and electrophysiological findings. The laboratory test results include complete blood count (lymphocytopenia: <20% of lymphocytes), serum sodium (hyponatremia: <135 mmol/L), serum potassium (hypokalemia: <3.5 mmol/L), serum albumin (hypoalbuminemia: <30 g/L), blood glucose level (hyperglycemia: fasting blood glucose ≥7.0 mmol/L or glycated hemoglobin ≥7.0%), liver function level and cerebrospinal fluid protein (g/L), and cell number (unit/mm^3^). And the tests of complete blood count, serum sodium, and serum potassium were examined in the 1st day of after admission, while the tests of serum albumin, fasting blood glucose, glycated hemoglobin, and liver function examined in the 2nd day of after admission. Generally we did lumbar puncture and electrophysiological examination around 2 weeks after onset. The results of electrophysiological examination were categorized into acute inflammatory demyelinating polyneuropathy, acute motor axonal neuropathy (AMAN), and unclassifiable group using the electrophysiological criteria proposed by Hadden et al.^[14]^

### 2.2 Clinical score and evaluation of short-term prognosis

Clinical scores of all the patients in these groups were evaluated at 2 time points (at nadir and at discharge) using HFGS and MRC score. The HFGS score was defined as follows:^[15]^ 0, healthy state; 1, minor symptoms and capable of running; 2, able to walk ≥5 m without assistance but able to run; 3, able to walk 5 m across an open space with help; 4, bedridden or chair-bound; 5, require assisted ventilation for at least a part of the day; and 6, dead. Muscle weakness was evaluated by the MRC sum score of 6 bilateral muscles in arms and legs, ranging from 0 (tetraparalytic) to 60 (normal strength).^[16]^ If the HFGS score was ≥4 points at nadir, it is regarded as severe GBS.^[17]^ Generally, the patient was allowed to discharged from the hospital when his condition was improved or stable in our department. Thus, if the HFGS score was ≥3 points when a patient was discharged from the hospital, the patient was considered to have a poor short-term prognosis.

### 2.3 Statistical analysis

Data were analyzed using the SPSS 17.0 software (IBM, West Grove, PA), and normality and homogeneity of variance tests were performed. The normally distributed measurement data were represented by mean ± standard deviation, and the means were compared using independent samples *t* test. Although the nonnormally distributed measurement data were represented by medians and interquartile ranges [M (Q1–Q3)] and were compared using the independent sample rank sum test. Chi-square test was used for evaluating the difference in the count data of patients in different groups. Logistic regression models were performed to determine risk factors of poor short-term prognosis. Variables that were statistically significant in univariate analysis were further analyzed in a multivariate regression analysis. For all statistical tests, *P* < 0.05 was considered to be significant.

## 3 Results

### 3.1 Distinct clinical features of GBS between elderly and nonelderly group

**Figure 1:**
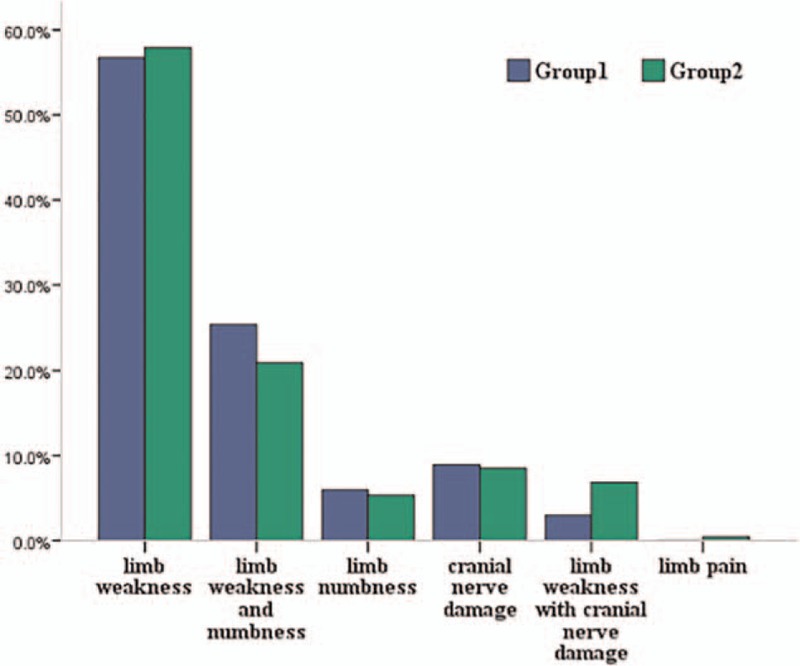
Comparisons of the initial symptoms between the 2 groups. Group 1 was patients aged ≥60 years while group 2 was patients aged 18 to 59 years. The initial symptoms of the patients in the 2 groups were as follows (group 1 vs group 2): limb weakness (56.7% vs 57.9%), limb weakness and numbness (25.5% vs 20.9%), limb numbness (6.0% vs 5.3%), cranial nerve damage (9.0% vs 8.5%), limb weakness with cranial nerve damage (3.0% vs 6.8%), and limb pain (0% vs 0.4%).

In total, 535 patients with GBS were enrolled in the study. There were 67 patients in the elderly group with a mean age of 69 years old, and 468 patients fell into the nonelderly group with a mean age of 39 years old. Higher proportion of male was found in both groups. The ratio of male to female in the 2 groups showed no significant difference (1.39:1 vs 1.64:1, *P* > 0.05). Comparisons of the clinical characteristics between the 2 groups were illustrated in Table 1. The incidence of antecedent infection in the elderly group was significantly lower than the nonelderly group (49.3% vs 66.2%, *P* < 0.01). Moreover, time from onset to admission (5 vs 4 days, *P* < 0.05) and time from onset to nadir in the elderly group (7 vs 6 days, *P* < 0.05) were both longer than the nonelderly group, indicating that the progression of elderly GBS patients was slower. Figure 1 revealed the comparisons of initial symptoms between the 2 groups and there was no statistically significant difference between the 2 groups (*P* > 0.05, excluding 2 patients with limb pain). In addition, the season of morbidity, tendon reflex, sensory dysfunction, cranial nerve damage, MRC score at nadir, HFGS score at nadir, the proportion of patients with severe GBS, and the proportion of patients requiring mechanical ventilation did not show statistically significant difference (*P* > 0.05).

**Table 1:**
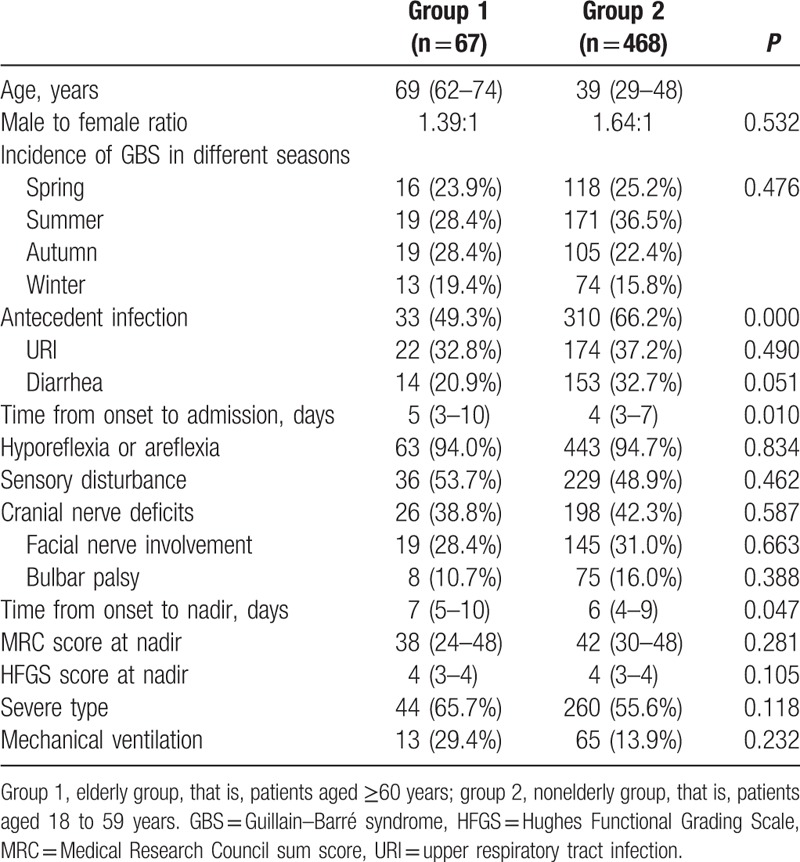
Comparison of clinical characteristics of GBS between elderly and nonelderly group.

### 3.2 Abnormal laboratory tests were more common in elderly patients with GBS

**Figure 2:**
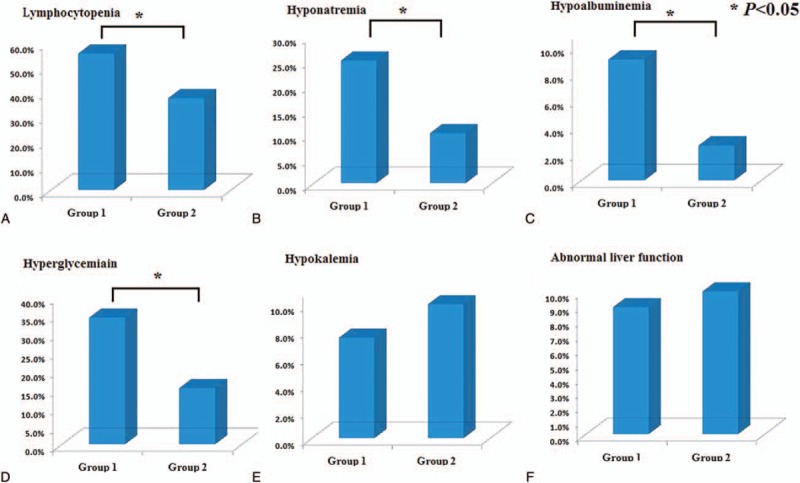
Comparisons of results of laboratory test. Group 1 was patients aged ≥60 years while group 2 was patients aged 18 to 59 years. The proportion of patients with lymphocytopenia was higher in group 1 than the group 2 (36/65 [55.4%] vs 167/448 [37.3%], *P* < 0.01) (A). The proportion of patients with hyponatremia was higher in group 1 than the group 2 (16/64 [25.0%] vs 44/432 [10.2%], *P* < 0.01) (B). The proportion of patients with hypoalbuminemia was higher in group 1 than the group 2 (6/67 [9.0%] vs 12/468 [2.6%], *P* < 0.05) (C). The proportion of patients with hyperglycemia was higher in group 1 than the group 2 (23/67 [34.3%] vs 71/468 [15.2%], *P* < 0.01) (D). The proportion of patients with hypokalemia showed no statistically significant difference in the 2 group (5/64 [7.8%] vs 47/440 [10.7%], *P* > 0.05) (E). The proportion of patients with abnormal liver function showed no statistically significant difference in the 2 group (5/64 [7.8%] vs 47/440 [10.7%], *P* > 0.05) (F).

We further compared the laboratory tests between the elderly and nonelderly groups. We found the proportion of lymphocytes in the elderly group was significantly lower than the nonelderly group (20.0% ± 10.0% vs 23.0% ± 10.4%, *P* < 0.05), and patients with lymphocytopenia were more common in the elderly group (Fig. 2A). Similarly, the proportion of patients with hyponatremia, hypoalbuminemia, and hyperglycemia was all higher in the elderly group than the nonelderly group (Fig. 2B–D). The incidence of hypokalemia and abnormal liver function in the 2 groups showed no statistically significant differences (Fig. 2E and F). Cerebrospinal fluid protein (0.76 [0.57–1.23] vs 0.81 [0.53–1.26], *P* > 0.05) (reference value: 0.15–0.45 g/L) and the cell count (3 [2–6] vs 4 [2–7], *P* > 0.05) also showed no statistically significant differences (40 patients in the elderly group and 292 patients in the nonelderly group received lumbar puncture). As to the results of the electrophysiological examination, we found no statistically significant differences between the 2 groups in Table 2 (41 patients in the elderly group and 213 patients in the nonelderly group received the electrophysiological examination).

**Table 2:**
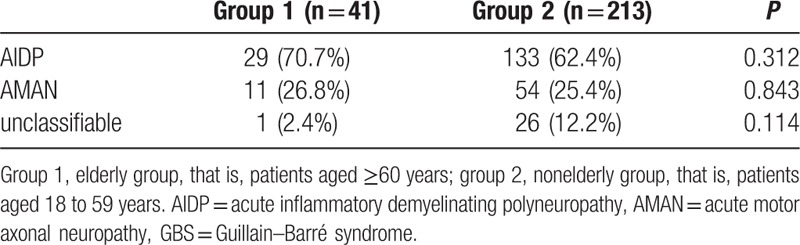
Comparison of electrophysiological examination of GBS between elderly and nonelderly group.

### 3.3 Elderly patients with GBS had poorer short-term prognosis

No statistically significant difference was observed in disease severity between the elderly and nonelderly groups. Similarly, the treatment modality did not differ between the 2 groups (Table 3). However, the duration of hospitalization was significantly longer in the elderly group (17 vs 14 days, *P* < 0.05), along with higher proportion of patients with pneumonia (29.9% vs 18.8%, *P* < 0.05) and poorer short-term prognosis at discharge from the hospital (58.2% vs 42.7%, *P* < 0.05) (Table 3).

**Table 3:**
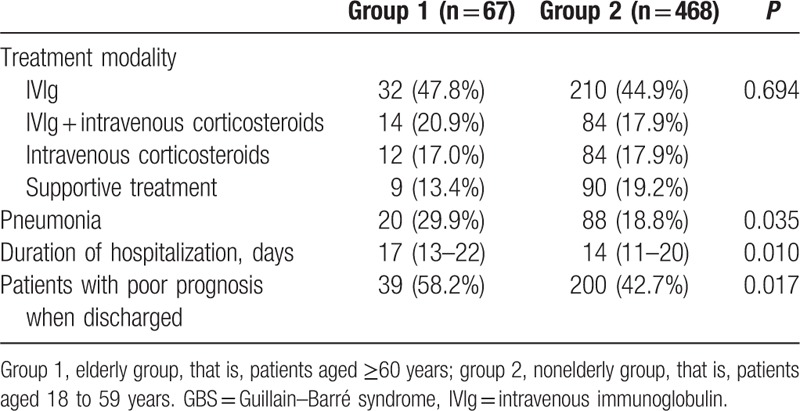
Comparison of short-term prognosis of GBS between elderly and nonelderly group.

### 3.4 Comparable short-term prognosis of GBS patients with a HFGS < 4 points between elderly and nonelderly group

Out of the 231 patients whose HFGS score < 4 points at nadir, patients in the elderly group had higher proportion of pneumonia (Table 4); however, no statistically significant differences were observed with regard to the factors such as disease severity (MRC and HFGS at nadir), treatment modality, duration of hospitalization, and the proportion of patients with poor prognosis at discharge from the hospital. No statistically significant differences were observed in the improvement of HFGS and MRC score between the 2 groups when patients discharged from the hospital (Table 4).

**Table 4:**
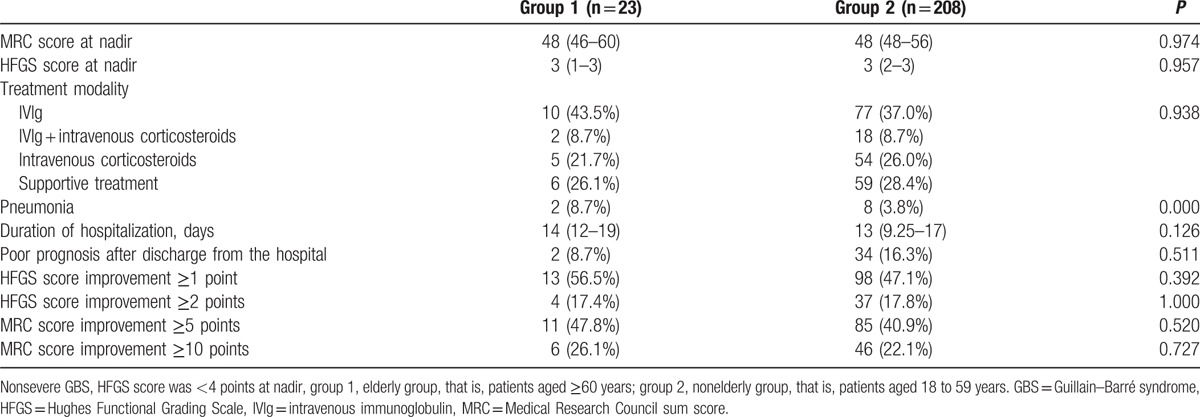
Comparison of hospitalization and discharge conditions of nonsevere GBS between elderly and nonelderly group.

### 3.5 Old age and lower nadir MRC serve as predictors for poor short-term prognosis in severe GBS patients

Totally, 304 patients with severe GBS were enrolled. Although no statistically significant differences were observed in disease severity, treatment modality, pneumonia, and the duration of hospitalization, the proportion of elderly patients with poor prognosis at discharge from the hospital was still higher (84.1% vs 63.8%, *P* < 0.01) (Table 5). Further analysis showed the proportion of patients with an improvement in HFGS score ≥2 and patients with an improvement in MRC score ≥10 from nadir to discharge were lower in the elderly group (Table 5). By using univariate analysis on variables including old age, antecedent infection, involvement of cranial nerve, MRC score at nadir, whether requiring mechanical ventilation, pneumonia, lymphocytopenia, hyponatremia, hypokalemia, hypoalbuminemia, hyperglycemia, abnormal liver function, axonal damage, and duration of hospitalization, we found that old age (≥60 years) (OR = 2.993, 95% CI: 1.284–6.979, *P* < 0.05), lack of antecedent infection (OR = 2.130, 95% CI: 1.244–3.646, *P* < 0.01), lower MRC score at nadir (OR = 0.949, 95% CI: 0.930–0.967, *P* < 0.01), pneumonia (OR = 1.840, 95% CI: 1.072–3.158, *P* < 0.05), and longer of duration of hospitalization (OR = 1.019, 95% CI: 1.001–1.036, *P* < 0.05) were associated with poor short-term prognosis. Furthermore, old age (OR = 2.906, 95% CI: 1.174–7.194, *P* < 0.05) and lower MRC score at nadir (OR = 0.948, 95% CI: 0.927–0.969, *P* < 0.01) were identified to be risk factors of poor short-term prognosis by multivariate analysis.

**Table 5:**
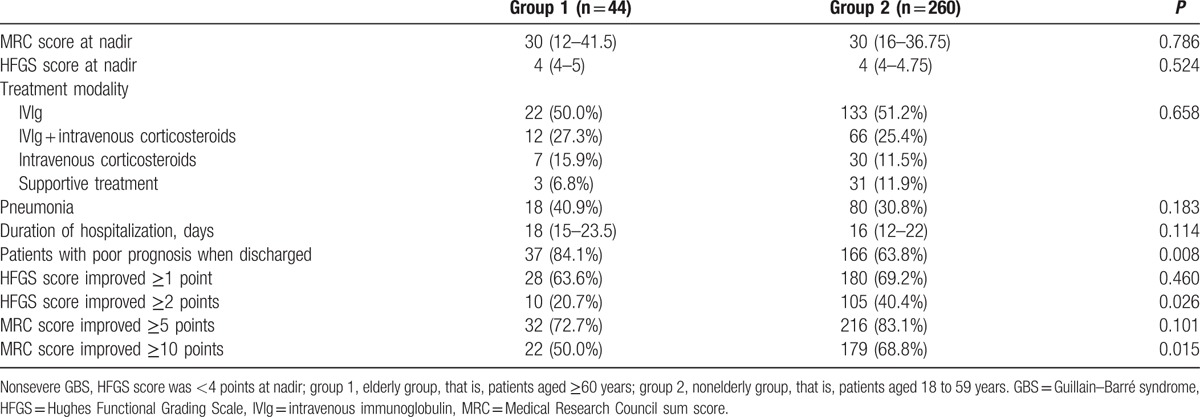
Comparison of hospitalization and discharge conditions of severe GBS between elderly and nonelderly group.

## 4 Discussion

In our study, we found that the elderly patients had slower disease progression, lower incidence of antecedent infection, higher incidence of abnormal laboratory tests, and poorer short-term prognosis. Further, old age and lower MRC score at nadir were found to be risk factors for poor short-term prognosis in severe GBS.

In general, GBS is a monophasic disease, which usually reaches the nadir within 4 weeks. GBS could occur at any age. In this study, we found that the disease progression in the elderly patients was slower, which was contradictory to Winner et al^[7]^ and Peric et al,^[9]^ who found that the time from onset of disease until admission/nadir was similar in the elderly patients and nonelderly patients. However, the disease severity of patients in the 2 groups showed no significant difference in the MRC score, HFGS score, severe type, and proportion of mechanical ventilation in our study. This finding was consistent with the results of Winner et al^[7]^ in UK, which found that the severity of disease was similar between elderly and nonelderly adults.^[7]^ However, our finding was contradictory to the results of Peric et al^[9]^ in Eastern Europe, which found that elderly patients had more severe disease. In this study, the disease progression and the severity of disease of elderly GBS patients were different from other countries’ findings, and it may be the peculiar feature of elderly GBS in China. Further, the epidemiologic studies of the different provinces in China are warranted to confirm this hypothesis.

GBS is considered as a disease caused by dysimmunity after infection, and there is evidence of antecedent infections in about two-thirds of the patients.^[1]^ It is of noteworthy that some other studies considered that immunosuppression may play an important role during the course of the disease^[3,18]^ and patients with noninfectious triggers may be associated with the immunocompromised states.^[1]^ In this study, the proportion of patients without antecedent infection and with lymphocytopenia in the elderly group was significantly higher than that in the nonelderly group, implying that immunosuppression may be associated with the occurrence of GBS in the elderly patients. These results of this study were consistent with the results of an earlier related study that elderly patients had less antecedent infection.^[19]^ In addition, we found that the elderly patients were prone to develop pneumonia during the course of the disease, which may be related to lymphocytopenia because lymphocytopenia would lessen the resistance to infection thereby increasing the risk of pneumonia. Some viral infections may induce lymphocytopenia; however, we could not confirm the relationship between lymphocytopenia and some certain viral infections in this study because we did not perform microbial or serological analyses. Meanwhile, classification of lymphocytes, detection of cytokines, and dynamic observation of changes in lymphocytes in our study were not done. We could not comprehensively evaluate the change of immune function for the patients. And further studies are required to further elucidate the immune mechanisms.

Importantly, we found that the elderly patients had poorer short-term prognosis than nonelderly patients, especially patients with severe GBS. This finding was further proved by the multivariate regression analysis, which identified old age and lower MRC score at nadir were independent risk factors for poor short-term prognosis in severe GBS patients. This result was consistent with previous studies which found that old age was a factor in predicting adverse prognosis of GBS.^[10–12,20,21]^ The poorer short-term prognosis of the elderly patients in our study might be due to the higher incidence of complications during hospitalization, such as lymphocytopenia, hyponatremia, hypoalbuminemia, hyperglycemia, and pneumonia. The proportion of hyponatremia in the elderly group was higher than that in the nonelderly group, which is consistent with previous studies.^[22,23]^ The hyponatremia may be due to the syndrome of inappropriate secretion of antidiuretic hormone, renal salt-wasting syndrome as part of dysautonomia, use of immunoglobulin, etc.^[23]^ However, we could not confirm the certain cause of hyponatremia in the study because we had not examined the antidiuretic hormone, the urine volume, and blood volume of the patients. And hyponatremia was found to increase the risk of death in a study.^[22]^ A higher proportion of hypoalbuminemia was also found in the elderly patients, which might increase the probability of infection and existing venous thrombosis.^[24]^ In addition, a higher proportion of hyperglycemia was found in the elderly patients. A previous study have suggested that diabetes might influence the prognosis of patients with GBS for 3 months.^[25]^

As to the severe GBS, except for old age and lower nadir MRC score, the univariate regression analysis showed that lack of antecedent infections, pneumonia, and longer duration of hospitalization were also relevant to poor short-term prognosis in patients with severe GBS. Lack of antecedent infection was related to poor short-term prognosis, which is consistent with the previous result that lack of antecedent infection is a risk factor for poor short-term prognosis in GBS patients with mechanical ventilation,^[26]^ as well is consistent with the results of Lin et al^[27]^ in Taiwan. Pneumonia was related to poor short-term prognosis, which is consistent with the result of a previous study that pneumonia was associated with duration of mechanical ventilation for GBS patients admitted to the intensive care unit.^[28]^ It may be an reason that the severity of illness prolonged the time of hospitalization, and longer duration of hospitalization might be related to poor short-term prognosis with severe GBS. McKhann et al^[29]^ found that the clinical features and prognosis of AMAN was different from acute inflammatory demyelinating polyneuropathy in 1993, while in this study we did not find the correlation between the short-term prognosis and the axonal damage (AMAN) in severe GBS patients. Electrophysiological findings might have prognostic relevance, but the results of different studies show variation.^[3]^ Also serial electrophysiological examinations will find some AMAN are characterized by reversible conduction failure,^[30]^ and these patients will recover good usually. Further studies are required to investigate the relationship between electrophysiological findings and prognosis in GBS by sequential electrophysiological examinations.

This study has following limitations. It was a retrospective study, especially the prognosis was done mainly on the patients of the hospital, lacking of follow-up observations to study the long-term prognosis. Also the detail data about autonomic nervous system involvement was not recorded in this study. We did not perform microbial or serological analyses in this study so that we could not confirm particular antecedent infection and might omit some subclinical antecedent infections. As we aimed to investigate the clinical features of elderly patients with GBS and we just made a retrospective study in The First Hospital of Jilin University, the incidence of GBS in nearby provinces was not investigated in the current study.

In summary, the elderly patients may have slower disease progression, lower incidence of antecedent infection, higher incidence of abnormal laboratory tests, and poorer short-term prognosis. Further, old age and lower MRC score at nadir are risk factors for poor short-term prognosis in severe GBS.
